# Vascular function in asthmatic children and adolescents

**DOI:** 10.1186/s12931-016-0488-3

**Published:** 2017-01-17

**Authors:** Leonardo Silva Augusto, Grazielle Caroline Silva, José Felippe Pinho, Rosária Dias Aires, Virgínia Soares Lemos, Lidiana Fátima Correa Ramalho, Nulma Souto Jentzsch, Maria Glória Rodrigues-Machado

**Affiliations:** 1 Faculdade Ciências Médicas-Minas Gerais, Pós-Graduação em Ciências da Saúde, 30130-110 Alameda Ezequiel Dias 275-Centro, Belo Horizonte, MG Brazil; 2 Departamento de Fisiologia e Biofísica da Universidade Federal de Minas Gerais, Belo Horizonte, MG Brazil; 3 Departamento de Medicina, Faculdade Ciências Médicas-Minas Gerais, Belo Horizonte, MG Brazil; 4 Unidade de Referência de Saúde Saudade, Prefeitura de Belo Horizonte (URS Saudade- SUS BH), Belo Horizonte, MG Brazil

**Keywords:** Endothelial dysfunction, Arterial stiffness, Reactive hyperemia index, Pulmonary function test, Exercise functional capacity, Quality of life

## Abstract

**Background:**

Epidemiological studies have demonstrated an increased incidence of cardiovascular events in patients with bronchial asthma, but little is known about the relationship between asthma and vascular function. The purpose of this study was to evaluate endothelial function and arterial stiffness in children and adolescents with asthma.

**Methods:**

A cross-sectional controlled study was designed. Measurements of endothelial function and arterial stiffness in asthmatic (13.6 ± 0.6 years) and control groups (14.9 ± 0.7 years) were taken by the non-invasive peripheral arterial tonometry (EndoPAT2000) determined by using the natural logarithm of the reactive hyperemia index (LnRHI) and the augmentation index (AIx@75%), respectively. Patients with asthma were also administered two questionnaires to evaluate asthma control and quality of life. Exercise functional capacity was evaluated using the Shuttle Walking Test (SWT). Only male participants were included in the present study.

**Results:**

LnRHI and the walked distance during the SWT were similar between groups (*p* = 0.23 and *p* = 0.50, respectively). AIx@75% was significantly higher in the asthmatic group (-7.75 ± 1.7) compared to the control group (-15.25 ± 1.8), *p* < 0.04. In the control group, the LnRHI correlated positively with baseline systolic blood pressure (*r* = 0.53, *p* = 0.02) and mean arterial pressure (*r* = 0.50, *p* = 0.03), age (*r* = 0.61, *p* = 0.007), weight (*r* = 0.63, *p* = 0.004) and height (*r* = 0.56, *p* = 0.015). Besides that LnRHI correlated with FVC (*r* = 0.69, *p* = 0.002), FEV_1_, (*r* = 0.53, *p* = 0.03) and negatively with Tiffeneau index (FEV_1_/FVC%, *r* = −0.49 *p* = 0.04). The LnRHI of the asthmatic group did not correlate with the different variables evaluated.

**Conclusion:**

The increased AIx@75% without changes in LnRHI in asthmatic patients could mean that an early detection of vascular impairment may precede endothelial dysfunction, and that different mechanisms may contribute to the pathogenesis and progression of cardiovascular events in this population. A large prospective and randomized controlled study should be done to evaluate the physiopathological mechanisms underlying the association between arterial stiffness and asthma.

**Electronic supplementary material:**

The online version of this article (doi:10.1186/s12931-016-0488-3) contains supplementary material, which is available to authorized users.

## Background

Asthma is a chronic inflammatory disease with a high prevalence that affects different age groups, especially children [1]. The inflammation that occurs in asthma patients is not restricted to the lungs. There is also systemic inflammation, which is characterized by an increase in plasma of various pro-inflammatory cytokines, such as interleukin-6 (IL-6), tumor necrosis factor alpha (TNF-α) and C-reactive protein (CRP) [2, 3].

Epidemiological studies have shown that asthma patients have a higher rate of cardiovascular diseases than patients without asthma [4–7]. Recent studies show an important association between the chronic systemic inflammation in asthma, and arterial stiffness, which is considered a prognostic factor and independent predictor of cardiovascular events [8, 9]. In young adults and children with asthma, arterial stiffness is inversely correlated to the percentage of forced expiratory volume in the first second (FEV_1_%) [9, 10], which demonstrating that correlation between pulmonary function and vascular stiffness is not limited to aging patients [11]. Moreover, it was observed that arterial stiffness tends to increase with asthma severity [8].

Endothelial dysfunction is currently considered to be the first event in a series of inflammatory or immune diseases, such as vasculitis and atherosclerosis [12]. This observation, coupled with the knowledge that asthma is considered an important inflammatory disease, led us to hypothesize that endothelial function could be changed in this population. Several studies have been recently performed in children and adolescents to assess endothelial function in different diseases [13, 14]. However, as far as we know, no previous study has evaluated the relationship between asthma and vascular dysfunction measuring simultaneously the reactive hyperemia index (RHI) and the augmentation index (Alx), noninvasive markers of endothelial function and arterial stiffness, respectively.

In the current study, we used EndoPAT2000 that quantifies the endothelium-mediated changes in vascular tone, elicited by a 5-min occlusion of the brachial artery [15, 16]. The increase in blood flow measured by the fingertip-mounted probe plethysmography that occurs when the cuff is deflated, stimulates the production and release of endothelium derived nitric oxide and cause vasodilation [17, 18]. The dilation, manifested as reactive hyperemia, is captured by the EndoPAT2000 as an increase in the digital pulse volume amplitude, and the recordings are automatically analyzed by computer-assisted analysis software [19]. Differently, AIx is widely used as an index of pulse wave reflection, and has been demonstrated to be an independent predictor of a cardiovascular event. This index modulates the central pressure profile [20], increasing aortic systolic pressure and decreasing aortic diastolic pressure [21]. Lower AIx reflects better arterial elasticity [18]. In healthy children and adolescents, EndoPAT2000 is feasible and has excellent reproducibility, providing an easy and reliable means of assessing endothelial function in this population [22].

## Material and methods

Participants in the study were children and adolescents (*n* = 19) with a clinical and spirometric diagnosis of asthma, according to Global Strategy for asthma management and prevention [1]. The control group (n = 18) was composed of healthy children and adolescents with anthropometric characteristics similar to the experimental group. Only male participants were included in the present study. All experimental protocols were approved by local ethics committee (protocol number # 34878614.3.0000.5134, Comitê de Ética e Pesquisa da Faculdade Ciências Médicas – Minas Gerais – CEPCM-MG). All participants and parents or legal guardians signed the Informed Consent Form (ICF).

Endothelial dysfunction and arterial stiffness were assessed through peripheral arterial tonometry (PAT; EndoPAT2000, Itamar Medical, Caesarea, Israel), a noninvasive technology that captures a beat-to-beat plethysmographic recording of the fingers [15]. A pair of pneumatic sensors (*plethysmographic probes*) was placed on the index fingers of each hand. Baseline measurements were collected for 5 min. After this time the blood pressure cuff, located on the subject’s non-dominate arm, was inflated to 60 mmHg above baseline systolic or at least 200 mmHg of pressure for 5 min (occlusion period), confirmed by a reduction of the PAT tracing to zero. After the occlusion period, the cuff was quickly deflated to assess changes in digital pulse volumes, a consequence of reactive hyperemia, i.e., endothelium-dependent flow-mediated vasodilation. To calculate the RHI, the ratio of the results of the recovery (cuff deflated) and baseline periods for the test arm (A/B) was divided by the ratio of the results of the recovery and baseline periods for the control arm (C/D), and then multiplied by the baseline correction factor [RHI = (A/B)/(C/D) x baseline correction factor] (Additional file 1: Figure S3). According to the manufacturer’s recommendation, values below 1.67 or 0.51 for the RHI or its natural logarithm of the reactive hyperemia index (LnRHI), respectively, characterize endothelial dysfunction [23]. These values are still not available in the literature for the pediatric population. The Alx was automatically calculated by the EndoPAT2000, using the pulse waveform at rest as a reference. The Alx was defined as the pressure difference between the peak of the reflection wave (P2) and the peak of the systolic wave (P1), expressed as a percentage of P1 [AIx = (P2-P1)/P1×100]. For the final analysis, the Alx was normalized to a heart rate of 75 beats per minute (Alx@75%). LnRHI and Alx@75% were calculated using an automatic algorithm of the EndoPAT2000 (Software 3.1.2).

The assessment of asthma control and quality of life were performed using the Asthma Control Questionary-ACQ [24] and Pediatric Asthma Quality of Life Questionnaire-PAQLQ [25], respectively, both translated and culturally adapted to the Brazilian population [26, 27].

The assessment of functional capacity was performed using the Shuttle Walking Test (SWT) [28]. The test was stopped if a patient had unable to maintain the required speed or experienced dizziness, shortness of breath, or fatigue.

Variables were described as mean ± SD. The unpaired *t*-test was used to compare the pulmonary function test parameters, LnRHI, Alx@75% and SWT, between the groups. The Mann-Whitney test was used to compare the differences for non-parametric data between groups. The relationships between vascular dysfunction (LnRHI and Alx@75) and other variables (anthropometric data, pulmonary function test and cardiorespiratory variables) were done using Pearson correlation coefficient or Spearman when applied. Data analysis was performed with the software GraphPad Prism version 6.0. The significance level was set at *p* < 0.05.

## Results

Asthma control levels considered in this study were controlled (n = 7), partly controlled (*n* = 7) and uncontrolled (*n* = 5). Asthmatic and control groups were similar in terms of age, weight, height, BMI, and walked distance, evaluated by SWT. The Tiffeneau index and FEV_1_ (%) were significantly lower in the asthmatic group compared to the control group (Table 1). Six volunteers (31.57%) from the asthmatic group had a positive bronchodilator test with at least a 12% increase in the FEV_1_ in accordance to GINA, 2015 [29].

**Table 1:** Anthropometric data, pulmonary function test and walked distance evaluated by shuttle walking test

Variables	Control	Asthmatic	*P* value
Anthropometric data			
Age (years)	14.89 ± 0.68	13.58 ± 0.60	*p* = 0.15
Weight (Kg)	54.11 ± 3.06	51.79 ± 3.02	*p* = 0.59
Height (m)	1.64 ± 0.03	1.63 ± 0.03	*p* = 0.91
BMI (Kg/m^2^)	19.86 ± 0.73	19.13 ± 0.54	*p* = 0.42
Pulmonary function test			
FVC (L)	3.85 ± 0.24	3.55 ± .29	*p* = 0.46
FVC (% predicted)	107.6 ± 6.62	93.2 ± 5.23	*p* = 0.09
FEV_1_(L)	3.37 ± 0.22	2.78 ± 0.27	*p* = 0.10
FEV_1_(% predicted)	99.89 ± 5.28	82.69 ± 6.24	*p* = 0.04
FEV_1_/FVC %	87.89 ± 1.72	77.04 ± 3.08	*p* = 0.005
Hemodynamic data			
SAP(mmHg)	105.0 ± 2.02	100.9 ± 2.64	*p* = 0.23
DAP(mmHg)	65.83 ± 1.62	62.11 ± 1.96	*p* = 0.15
MAP(mmHg)	78.89 ± 1.45	75.75 ± 2.05	*p* = 0.22
HR(bpm)	74±3	80±2	*p* = 0.17
Shuttle walking test			
Walked distance(m)	470.6 ± 33.03	442.4 ± 25.37	*p* = 0.5

*FVC* forced vital capacity, *FEV*_*1*_ forced expiratory volume in the first second, *FEV*_*1*_ */FVC* Tiffeneau Index, *SAP* systolic arterial pressure, *DAP* diastolic arterial pressure, *MAP* mean arterial pressure, *HR* heart rate, *SWT* shuttle walking test

Based on the domains of symptoms (5.61 ± 0.26), emotions (5.83 ± 0.27), activities (5.32 ± 0.30) and the general score (5.59 ± 0.25) assessed by PAQLQ, there was a moderate loss of quality of life in the asthmatic patients.

**Fig. 1:**
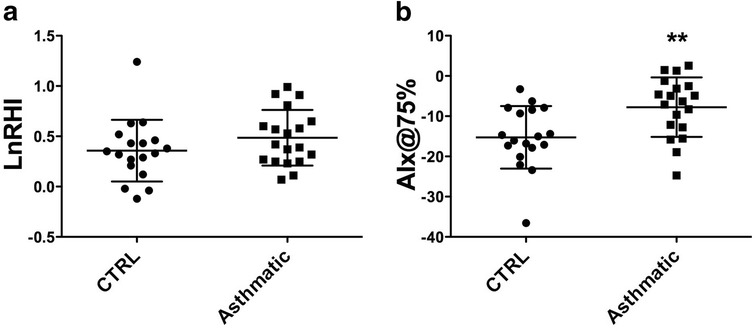
Vascular function, given by the, natural logarithm of the reactive hyperemia index (LnRHI) and the augmentation index corrected for a heart rate of 75 bpm (AIx@75%). **p < 0.001 compared to the control group

Figure 1 shows vascular function results. The LnRHI did not differ between the control and asthmatic groups (*p* = 0.23). However, the Alx@75% was significantly higher in the asthmatic group (*p* < 0.001). In the control group, the LnRHI correlated positively with baseline systolic blood pressure (*r* = 0.53, *p* = 0.02) and mean arterial pressure (*r* = 0.50, *p* = 0.03), age (*r* = 0.61, *p* = 0.007), weight (*r* = 0.63, *p* = 0.004) and height (*r* = 0.56, *p* = 0.015). Besides that LnRHI correlated with forced vital capacity (FVC, *r* = 0.69, *p* = 0.002), forced expiratory volume in the first second (FEV_1_, *r* = 0.53, *p* = 0.03) and negatively with Tiffeneau index (FEV_1_/FVC%, *r* = −0.49 *p* = 0.04). The LnRHI of the asthmatic group did not correlate with the different variables evaluated. (Additional file 1: Table S1).

## Discussion

As far as we know, no study has yet assessed the markers of endothelial function and arterial stiffness in asthmatic children and adolescents, using the EndoPAT2000. In this study we demonstrated that LnRHI did not differ between the control and asthmatic groups and the Alx@75% was significantly higher in the asthmatic group, suggesting that the early detection of arterial stiffness marker may precede endothelial dysfunction.

Long-term exposure to inflammation, even at low levels, triggers a series of events that promote alterations in the regulation of lipid metabolism, atherosclerosis and endothelial dysfunction, which are precursors of cardiovascular disease [30, 31]. According to Tattersall et al. [32], asthmatic patients present higher rates of cardiovascular disease compared to participants without asthma, suggesting that there is a shared pathophysiological element between asthma and cardiovascular disease, triggering and perpetuating different types of comorbidities. In the present study, we investigated the association between the endothelial and pulmonary functions in children and adolescents with asthma. Only male participants were included in the present study. There is a higher probability of male children to develop early vascular changes compared to female children of the same age [33] and there are differences in the reactive hyperemia indexes when comparing male and female volunteers [33]. Moreover, females present hormonal fluctuations during the menstruation period and the first years of puberty, which can interfere with measurements. It is also difficult to control the use of contraceptives in adolescents, another important factor that contributed to the endothelium dysfunction. Also, only participants with a normal BMI were included in the present study because overweight and obesity are factors that lead to endothelial dysfunction [34].

In the current study, the LnRHI of the asthmatic group did not differ from control group. Similar results were observed by Moore et al. [35] through flow mediated dilation (FMD) technique, based on the same physiological mechanism of PAT [15]. A main advantage of the EndoPAT2000 system on FMD is that the contralateral arm serves as an internal control of measurement [36] and this technique is operator-independent [36]. In addition, EndoPAT2000 enables the simultaneous measurement of endothelial function and arterial stiffness. In the current study, LnRHI of control group correlated positively with baseline systolic blood pressure and mean arterial pressure, anthropometric data (age, weight, height), and pulmonar function test parameters (FVC, FEV_1_). In addition, in control group the LnRHI correlated negatively with Tiffeneau index. A large sample sized, prospective and randomized controlled study should be addressed to evaluate the value of these associations.

According to Harkness et al. [37] asthma is not only an airway disease, but a vascular disease due to an expansion and morphological dysregulation of the bronchial vascular network in the airways of asthmatics, such as an increased number, size and density of blood vessels, as well as vascular leakage and plasma engorgement. Recently we demonstrated [38, 39] in a model of chronic asthma induced by ovalbumin (OVA) that asthmatic mice exhibited an increase in size and number of vessels within the airway wall, contributing to thickening of the wall. OVA mice also presented a significant increase in myocyte diameter of the right ventricle, suggesting that structural remodeling led to an increase in pulmonary vascular resistance and subsequent right ventricular hypertrophy. However, the pathophysiological impact of the increased vasculature in the bronchial wall and its significance to pulmonary function in asthma [37] and systemic circulation is unrecognized at this time.

Accumulating data have shown an important association between chronic systemic inflammation in asthma and arterial stiffness [9–11], which is considered a prognostic factor and an independent predictor of cardiovascular events [8]. In the present study, the Alx@75% was significantly higher in the asthmatic group but different from other authors [8–10], there was no correlation between this index and the pulmonary function parameters and reversibility of airway obstruction in the asthmatic group. This suggests that, besides airway obstruction, there are other factors contributing to the higher incidence of cardiovascular outcomes in this population. The results of Moore et al. [35] corroborates with this line of thought. Moore et al. [35] compared the arterial stiffness in asthmatic adults (18 to 45 years), having controlled and partially controlled asthma, with healthy volunteers, which were matched for age, body mass index, level of physical activity, and maximum oxygen consumption (VO_2_max). These authors observed that arterial stiffness was significantly higher in patients with asthma, suggesting that structural changes in arterial vessels are related to pulmonary disease and are not a consequence of reduced physical activity or VO_2_max. Similarly to Moore et al. [35] results, in the present study healthy volunteers were matched for age, high, body mass index, and exercise functional capacity.

Despite the exclusion of factors that could mislead the identification of endothelial dysfunction, such as female gender, changes in the BMI, smoking and diseases that can compromise endothelial function such as diabetes, this study has some limitations. The first limitation is the lack of knowledge about the lipid profile of the participants. Inflammatory and hemostatic abnormalities present in children with familiar hypercholesterolemia contribute to endothelial dysfunction [40]. Another limitation of this study was the lack of assessment of the pubertal stage of the participants. Endothelial function increases during puberty. Children at advanced stages of pubertal development, assessed by the scale of Tanner, have a greater peripheral vasodilator response measured by PAT [41]. Lastly, black volunteers were not excluded from the study. This ethnicity is usually associated with impaired vascular function compared to whites, due to the lower bioactivity of nitric oxide in the microcirculation of the forearm, which is associated with a reduced vasodilator response of the smooth muscle in response to nitric oxide donors [42].

In conclusion, this study demonstrated that LnRHI did not differ between the groups and the Alx@75% was significantly higher in the asthmatic group. The increase in Alx@75% in asthmatic patients, with no changes in LnRHI, may indicate that early detection of vascular dysfunction may precede endothelial dysfunction and that different mechanisms may contribute to the pathogenesis and progression of cardiovascular events in this population.

## Additional file

Additional file 1: Supplemental material. (DOCX 1048 kb)

## Abbreviations

ACQ: Asthma control questionnaire
AIx@75%: AIx normalized to a heart rate of 75 beats per minute
Alx: Augmentation index
BMI: Body mass index
BP: Blood pressure
CRP: C-reactive protein
FEV_1_: Forced expiratory volume in the first second
FVC: Forced vital capacity
HR: Heart rate
IL-6: Interleukin-6
LnRHI: Natural logarithm of the reactive hyperemia index (LnRHI)
MAP: Mean arterial pressure
PAQLQ: Pediatric asthma quality of life questionnaire
RHI: Reactive hyperemia index
RR: Respiratory rate
SWT: Shuttle walking test
TNF-α: Tumor necrosis factor alpha
