# Supplementary figures and images for: Vascular function in asthmatic children and adolescents
Source: Respir Res. 2017 Jan 17;18:17. doi: 10.1186/s12931-016-0488-3 (PMC5240276; doi:10.1186/s12931-016-0488-3)

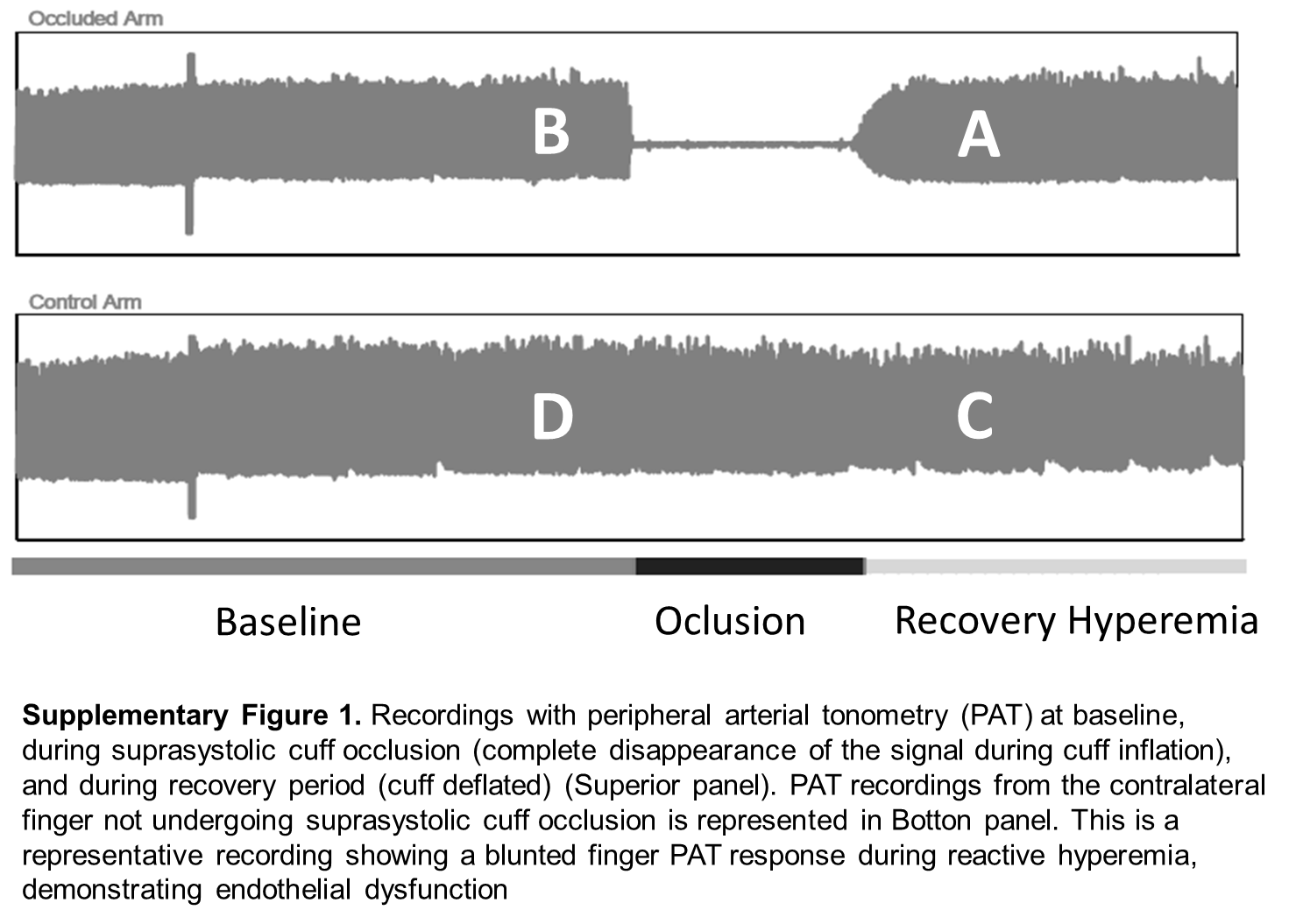


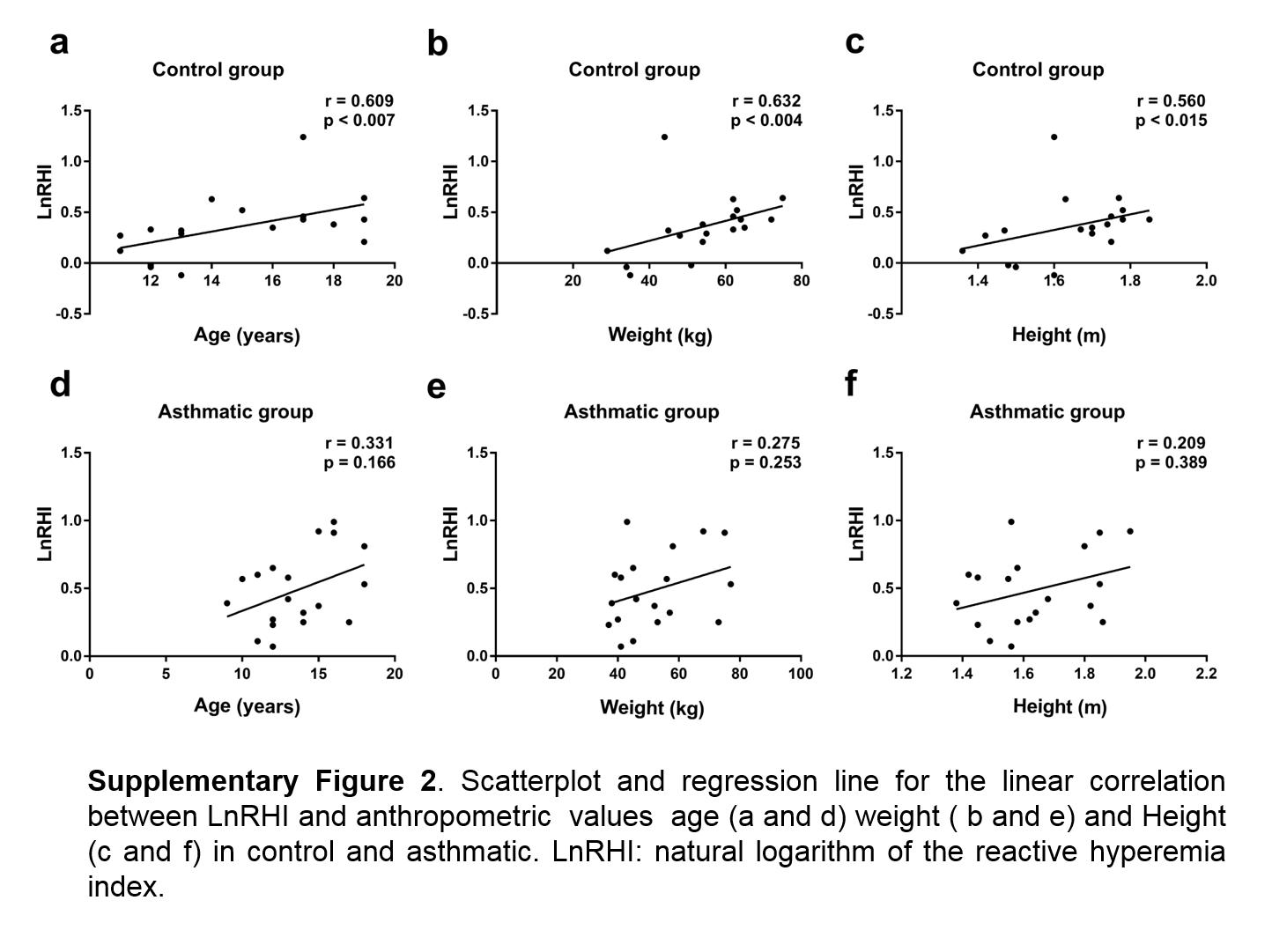


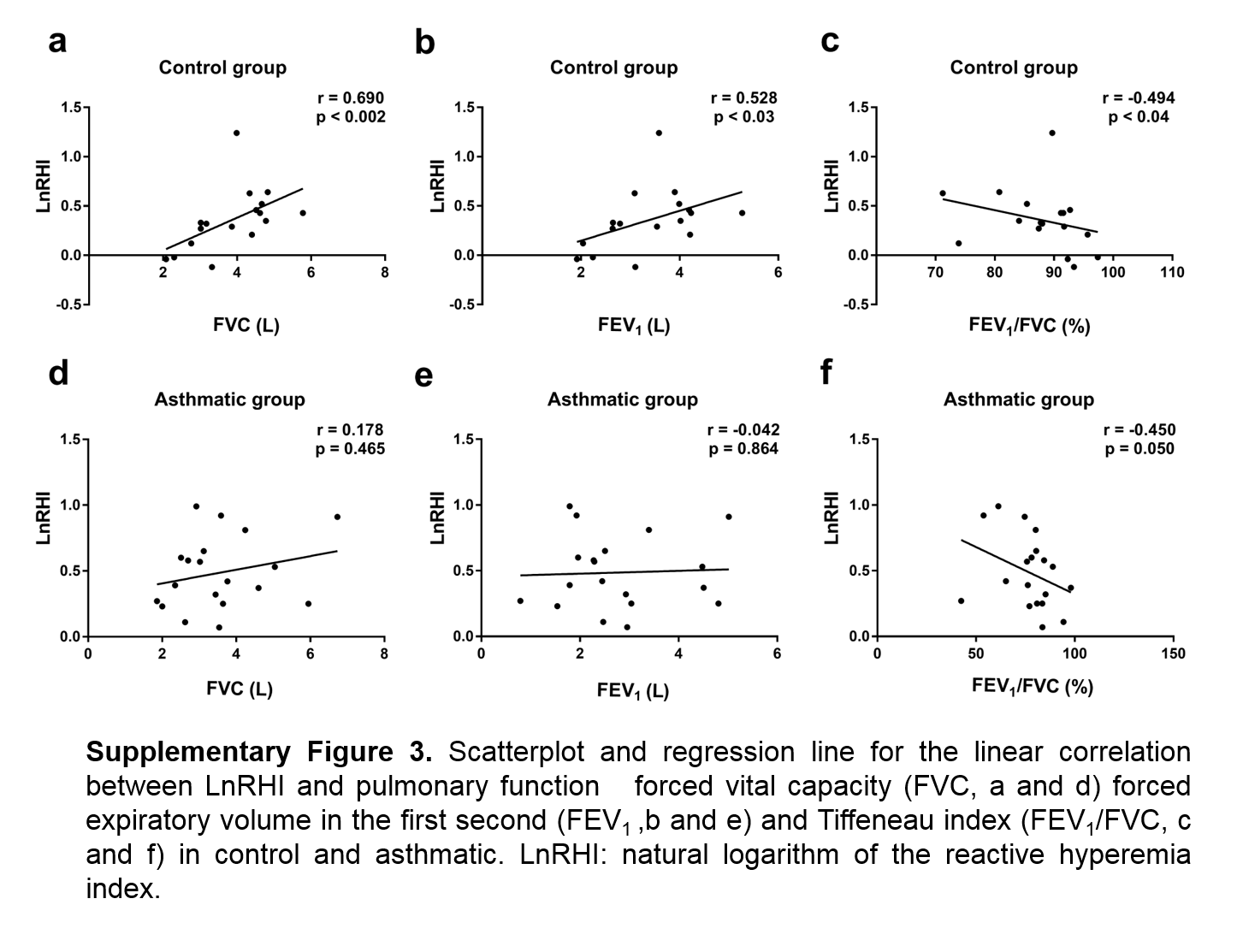


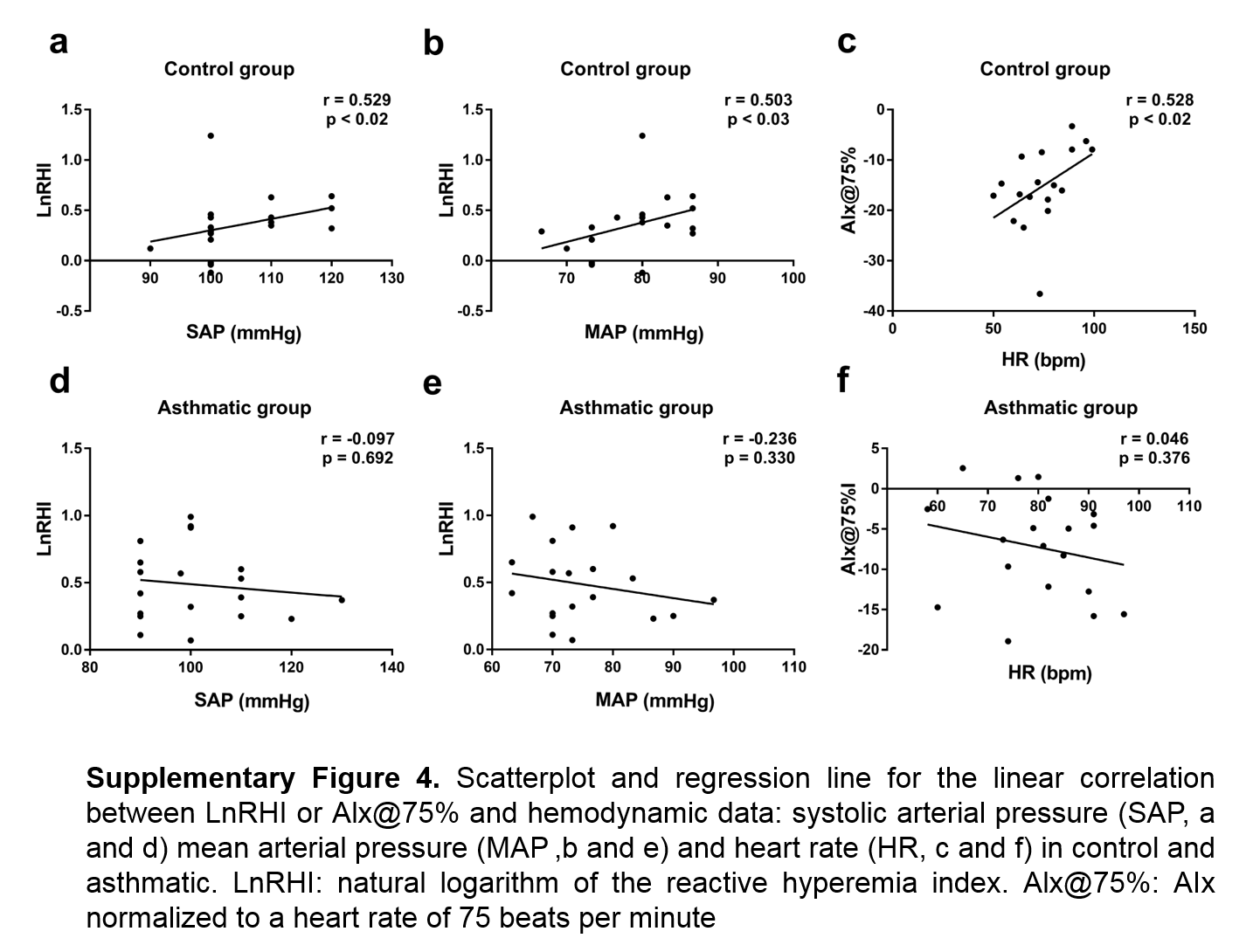

Supplement: Additional file 1: — Supplemental material. (DOCX 1048 kb) [file 12931_2016_488_MOESM1_ESM.docx]
